# Burnout in medical students: a systematic review of experiences in Chinese medical schools

**DOI:** 10.1186/s12909-017-1064-3

**Published:** 2017-11-16

**Authors:** Wang Michael Chunming, Reema Harrison, Raina MacIntyre, Joanna Travaglia, Chinthaka Balasooriya

**Affiliations:** 1 School of Public Health, University of New South Wales, Samuels Building, Sydney, NSW 2052 Australia; 2 Centre for Health Services Management, University of Technology Sydney, Sydney, Australia

**Keywords:** Medical education, Burnout, Emotional exhaustion, Depersonalisation

## Abstract

**Background:**

To identify the: extent to which medical students in China experience burnout; factors contributing to this; potential solutions to reduce and prevent burnout in this group; and the extent to which the experiences of Chinese students reflect the international literature.

**Methods:**

Systematic review and narrative synthesis. Key words, synonyms and subject headings were used to search five electronic databases in addition to manual searching of relevant journals. Titles and abstracts of publications between 1st January 1989-31st July 2016 were screened by two reviewers and checked by a third. Full text articles were screened against the eligibility criteria. Data on design, methods and key findings were extracted and synthesised.

**Results:**

Thirty-three studies were eligible and included in the review. Greater levels of burnout were generally identified in males, more senior medical students, and those who already experienced poorer psychological functioning. Few studies explored social or contextual factors influencing burnout, but those that did suggest that factors such as the degree of social support or the living environment surrounding a student may be a determinant of burnout.

**Conclusions:**

Greater understanding of the social and contextual determinants of burnout amongst medical students in China is essential towards identifying solutions to reduce and prevent burnout in this group.

## Background

Burnout amongst health professionals is characterised by *“various degrees of emotional exhaustion, depersonalization and a low sense of personal accomplishment”* [1]. Although the detrimental consequences of burnout on clinician well-being and patient care are widely documented, burnout continues to be endemic in the health system [2–9].

Symptoms of burnout are prevalent from the outset of medical training, with multi-institutional studies indicating that at least 50% of medical students may meet burnout criteria at some point during their studies [6–18]. Burnout in medical school has potential to negatively impact on students’ academic development and overall well-being, with burnout identified as a significant independent predictor of suicide ideation and dropping out of medical school [11]. A number of potential solutions and strategies to prevent and reduce burnout amongst medical students are evidenced in literature, but these largely report data solely from students in English-speaking countries.

Strategies at individual, and structural or organisational strategies have all reported clinically meaningful reductions in burnout amongst doctors [19]. Such strategies include the: promotion of healthier lifestyle choices and social activities in medical training; provision of psychosocial support; web-based interventions; guidance to support positive attribution styles during the teaching process; and elective courses for learning relaxation techniques [20–24]. In the workplace, contextual factors such as working hours, social support and relationships with co-workers have been increasingly recognised as key determinants of burnout and therefore targets for intervention [25]. Yet a recent systematic review highlighted that knowledge regarding the interventions that are most effective for specific populations of medical students is lacking [19].

China has the largest number of medical practitioners in the world, and these practitioners service the world’s biggest population of 1,400,000,000, yet studies that report the experiences of medical students in China are under-represented in the English language literature [26]. As such, there is a dearth of knowledge about the contextual factors that influence burnout in Chinese medical students. It is important to explore this area in order to understand not only the scale of the problem of burnout in Chinese medical students but also to develop possible evidenced-based solutions.

To address this gap, a systematic review of studies of burnout amongst medical students in China in both mainstream and Chinese language research databases, was undertaken. The review had four aims. These were to identify: 1) the extent to which medical students in China are experiencing burnout; 2) the demographic, social and psychological factors contributing to this, 3) potential solutions to reduce and prevent burnout in China and; 4) the extent to which experiences in China reflect the international literature.

## Methods

The Preferred Reporting Items for Systematic Reviews and Meta-Analyses (PRISMA) statement is an evidence-based approach for reporting in systematic reviews and meta-analyses. The PRISMA statement was used to guide the reporting of this systematic review [27].

A range of text words, synonyms and subject headings were developed for the three major concepts in this review of 1) burnout, 2) the associated constructs of emotional exhaustion, depersonalisation and personal accomplishment, and 3) medical education settings in China. These phrases were combined with AND and used to undertake a systematic search of five electronic databases from January 1989 to July 2016. The date range was selected to identify sufficient relevant literature within a relatively recent timeframe given the changing context of medical education. Databases searched were: MEDLINE, China Academic Journals Full-text Database, Chinese Scientific Journals Database,Wanfang Data Resource System. Hand searching of relevant journals (e.g. Chinese Journal of Medical Education Research and Chna Higher Medical Education) and reference lists of the included papers ensured that relevant published material was captured. Results were merged using reference-management software (Endnote) and duplicates removed.

Several limitations were applied. Only studies with the following.

characteristics were included: available in English or Chinese languages that reported original primary data published from January 1989–July 2016; subjects were medical students of Chinese origin studying in China; any study design (including quantitative, qualitative and mixed-methods research); any study which validated or purpose-developed assessment of burnout or its constructs of emotional exhaustion, depersonalisation or personal accomplishment. In studies that included other professions in the sample, only data relating to medical students were extracted for the present review.

Articles were excluded if they did not meet the above criteria. Literature assessing hypothetical vignettes or scenarios rather than actual experience was excluded, in addition to studies that focused on general well-being or psychological well-being but not specifically burnout. Articles that focus on medical teachers, trainees, residents, nurses and nurse students were excluded.

### Study selection and data extraction

Two reviewers independently screened the titles and abstracts (WC; CB). Copies of full articles were obtained for those that were potentially relevant. Inclusion criteria were then independently applied to the articles by the two reviewers. Disagreements were resolved by consensus or consultation with a third reviewer. The following data were extracted: author(s), publication year, sample, setting, design, primary focus and main findings.

### Data synthesis

Findings were analysed using a narrative synthesis in stages based on the study objectives [28]. A narrative approach was utilised to synthesize the findings as given the heterogeneity of the outcome measures used, whilst many studies used variants of the same measure, these were not consistently used in every study and therefore a narrative synthesis was appropriate on this occasion. A quantitative approach was not considered appropriate as the measures were not directly comparable [28]. Initial descriptions of the eligible studies and results were tabulated (presented in Table 1). Patterns in the data were explored to identify consistent findings in relation to the study objectives. Interrogation of the findings explored relationships between study characteristics and their findings; the findings of different studies; and the influence of the use of different outcome measures, methods and settings on the resulting data.

**Table 1:** Summary of included studies (*n* = 33)

Lead author	Year	Participants	Outcome measure	Items	Primary aim	Key findings
Chen [39]	2012	492 postgraduate medical students	MBI-GS	15	• To determine burnout levels and related factors.	• 38.4% students had moderate or high levels of burnout. • There was a correlation between burnout level on the dimension of EX and age, marriage status, course (PHD/Master), working hours, coping styles, anxiety. • There was a correlation between burnout level on the dimension of CY and age, type of course, coping styles, anxiety. • There was a correlation between burnout level on the dimension of PE reduction and gender, coping styles and anxiety.
Chen [44]	2011	471 undergraduate medical students	LRS	20	• To determine burnout levels and related factors.	• 30.6% students had above moderate levels of burnout. • Male students suffered more burnout on the dimension of EX. • There was a significant correlation between burnout and student-origin on the dimension of EX and CY. Students from cities suffered more burnout.
Shen [40]	2012	111 postgraduate medical students	LRS	20	• To determine burnout levels and correlation with the sense of professional commitment.	• The level of burnout among postgraduate medical students was average (not serious). • There was a correlation between overall burnout level and the sense of continuing commitment.
Di [37]	2014	635 undergraduate medical students	LRS	20	• To determine burnout levels and related factors.	• 41.7% students had above moderate levels of burnout. • Male students suffered more burnout than females on the dimensions of EX and CY. • Social support and professional commitment were negatively correlated to burnout.
Fan [52]	2015	277 undergraduate medical students	MBI-SS	Not stated	• To determine the correlation between burnout levels and occupational motivation.	• There was no significant correlation between burnout level and occupational motivation.
Fu [61]	2012	131 undergraduate medical students; 119 non-medical students	LRS	20	• To test the difference between burnout levels in medical students and non-medical students.	• Non-medical students had more serious burnout than medical students.
Hu [49]	2014	866 undergraduate medical students	Li et al. 2011 Burnout Scale	Not stated	• To determine burnout levels and related factors.	• There was significant difference of burnout level on the dimension of EX by different grades.
Jiang [38]	2008	42 non’211′ university students; 38 ‘211′ university students	MBI-GS	15	• To determine burnout levels, related factors and differences between students from ‘211’ and non ‘211’ universities.	• Burnout level of students from non’211′ university are higher than ‘211′ on the dimension of EX and CY. • There is negative correlation between burnout and organizational justice
Jin [43]	2010	77 undergraduate and postgraduate medical students	MBI-GS	15	• To determine burnout levels and correlation with psychological problems and symptoms of psychopathology.	There was no significant correlation between burnout and psychological problems and symptoms of psychopathology.
Li [62]	2015	1220 postgraduate medical students	MBI-HSS	22	• To test burnout levels.	• The burnout level on the dimensions of CY and PE reduction were more serious comparing to the MBI-HSS norm score
Li [63]	2015	224 postgraduate medical students	MBI (version not stated)	Not stated	• To determine burnout levels and related factors.	• Students’ level of burnout was moderate. • There was a significant correlation between burnout level and gender, marital status, drinking, smoking, student-origin.
Li [42]	2009	120 postgraduate medical students; 102 interns	MBI-GS	22	• To determine burnout levels and related factors.	• Students scored highly on the burnout dimensions of EX (39%) and PE reduction (52%) but less so on the dimension of CY (21%). • There was no significant difference between medical students and physicians on burnout levels. • Social support was associated with burnout levels on the dimension of EX and CY. • Medical students reported a lack of social support compared to physicians.
Li H [55]	2011	155 undergraduate medical students	MBI	22	• To determine burnout levels and related factors.	• There were significant correlations between burnout and the age and gender. The older the students the more serious burnout level on the dimension of CY. Male students suffered more than female students on the burnout dimension of CY.
Ling L [23]	2014	200 undergraduate medical students	LRS	20	• To explore burnout levels and their correlation with parents’ upbringing style.	• There was a significant correlation between burnout level and parents’ upbringing style.
Li L [48]	2013	679 undergraduate medical students	LRS	20	• To determine burnout levels and related factors.	• Self-efficacy and attributional style were negatively correlated to burnout • Scholarship and grade were predictors of students’ burnout level.
Li YZ [64]	2014	137 western medicine undergraduate medical students; 123 traditional Chinese medicine undergraduate students	LRS	20	• To determine burnout levels and related factors. • To compare burnout levels between medical students learning western medicine and medical students learning traditional Chinese medicine.	• 25.8% students had above moderate levels of burnout. • Medical students learning traditional Chinese medicine suffered more burnout than medical students learning western medicine on the dimensions of CY and PE reduction • There was significant difference in burnout level on the dimension of CY by grade among medical students learning traditional Chinese medicine. Grade two suffered the most burnout on the dimension of CY.
Liao [36]	2011	627 undergraduate medical students	Scale based on LRS	24	• To determine burnout levels and related factors.	• 52.1% students reported above moderate level of burnout • There was a significant correlation between burnout and student grade on the dimension of EX. Students from higher grade suffered burnout more commonly. • There was a significant correlation between burnout and student-origin on the dimension of PE reduction. Students from cities suffered more burnout.
Lu [34]	2012	80 undergraduate medical students	MBI	Not stated	• To determine burnout levels.	• 41% students are above moderate level of burnout.
Song [54]	2015	144 undergraduate medical students	MBI-GS	15	• To determine the correlation between burnout levels and accomplishment motivation.	• There was a significant negative correlation between burnout and accomplishment motivation on the dimensions of seeking success motivation. • There is significant correlation between burnout and accomplishment motivation on the dimensions of avoiding failure motivation
Song [53]	2013	378 undergraduate medical students	Ni et al. 2009 Burnout Scale	14	• To explore burnout levels and their correlation with study motivation and psychological capital	• Study motivation and psychological capital were negatively correlated to burnout.
Sun [56]	2013	458 undergraduate medical students	MBI	Not stated	• To explore burnout levels and their correlation with social support and self-efficacy	• Social support and self-efficacy were negatively correlated to burnout.
Wang [46]	2011	312 undergraduate medical students	Ni et al. 2009 Burnout Scale	14	• To determine burnout levels and related factors.	• The level of burnout among medical students is above average (serious). • There were significant correlations between burnout and gender and burnout and grade. Male students suffered more than female students on the burnout dimension of CY, but less on the burnout dimension of PE reduction. Grade 2 and 3 students suffered more than grade 1.
Wei [51]	2014	748 undergraduate medical students	LRS	20	• To explore burnout levels and their correlation with study pressure.	• There was a significant correlation between burnout level and study pressure.
Wu [50]	2012	388 undergraduate medical students	LRS	20	• To determine burnout levels, related factors and correlation with the sense of professional commitment.	• Students’ level of burnout was reported as moderate. • There was a significant negative correlation between burnout and scores of exams. • There was a correlation between burnout level and the sense of professional commitment on all the dimensions including affective commitment, normative commitment, continuing commitment and ideal commitment.
Xiao [45]	2013	442 undergraduate medical students	LRS	20	• To test the correlation between burnout and emotional intelligence.	• Overall burnout was higher in males except in PE reduction dimension. • There was a significant negative correlation between burnout and emotional intelligence.
Xu^[35]^	2009	610 undergraduate medical students	LRS	20	• To determine burnout levels and organizational, social and individual factors related to this.	• 39.5% students were suffering burnout, especially on the dimension of CY. • Male students and more senior students suffered more burnout than junior or female counterparts. • Social support and learning environment were significant correlated with burnout.
Yang^[31]^	2013	952 undergraduate medical students; 299 undergraduate nursing students	LRS	20	• To explore burnout levels and their correlation with professional commitment and time management disposition.	• The burnout levels of medical students on the dimension of CY and PE reduction were less serious than nurse students. • Professional commitment was negatively correlated to burnout. • Professional commitment was correlated to and time management disposition.
Yang^[30]^	2011	576 undergraduate medical students	LRS	20	• To determine burnout levels and related factors and evaluate the effectiveness of group coaching intervention to deal with burnout among medical students.	• The level of burnout among medical students was moderate and influenced by grade, academic performance, student-origin, family parenting pattern. • The higher the pressure, the more serious the burnout. Problem-focused coping styles were identified as beneficial to reduce burnout. Emotion-focused coping styles were identified as detrimental. • Group Intervention reduced burnout effectively by enhancing coping styles.
Zhang^[47]^	2013	323 undergraduate medical Students	LRS	20	• To explore the correlation between burnout and the dormitory environment.	• There was a significant negative correlation between burnout on the dimension of EX and dormitory environment on the dimension of social environment and learning environment. • There was a significant negative correlation between burnout (dimension of CY) and dormitory environment (dimension of social environment and learning environment).
Zhang^[57]^	2011	642 undergraduate medical students; 628 non-medical students; 132 physicians	MBI-HSS	22	• To determine burnout levels and related factors.	• Burnout levels were higher amongst medical students than non-medical students, and higher than physicians on the dimension of PE reduction, but lower than physicians on the dimensions of EX and CY. • There was a significant correlation between burnout and psychological problems and symptoms of psychopathology.
Zheng^[41]^	2015	545 postgraduate medical students	LRS	20	• To determine burnout levels and related factors.	• There was a significant difference of burnout level on the dimension of EX by different grades. Grade three students were the most burned out. • Male students suffered more burnout than female students on the dimension of EX.
Zhu HC^[32]^	2012	87 postgraduate medical students	MBI-GS	Not stated	• To determine burnout levels and correlation with psychological problems and symptoms of psychopathology and the other factors.	• 71.1% students are above moderate level of burnout. • Male students scored higher on the dimension of EX. • There is significant correlation between burnout and psychological problems and symptoms of psychopathology.
Zhu Y^[33]^	2012	184 undergraduate or postgraduate medical students	LRS	20	• To determine burnout levels and correlation with exam performance.	• 37.5% students are above moderate level of burnout. • Male students suffer more overall burnout than females. • There was a significant negative correlation between burnout and scores of exams.

### Data appraisal

All the papers were evaluated using medical education research study quality instrument (MERSQI) [29]. The possible score of MERSQI range 5–18, including study design (1–2), sampling (1–3), type of data (1–3), validity of evaluation instruments’ scores (0–3), data analysis (1–3), outcome (1–3). Two reviewers individually assessed all publications; disagreements were resolved through discussion. Due to the limited number of eligible publications, we did not exclude studies based on the quality assessment; quality assessment data was used simply to portray the strength of the available evidence.

## Results

### Search results

**Fig. 1:**
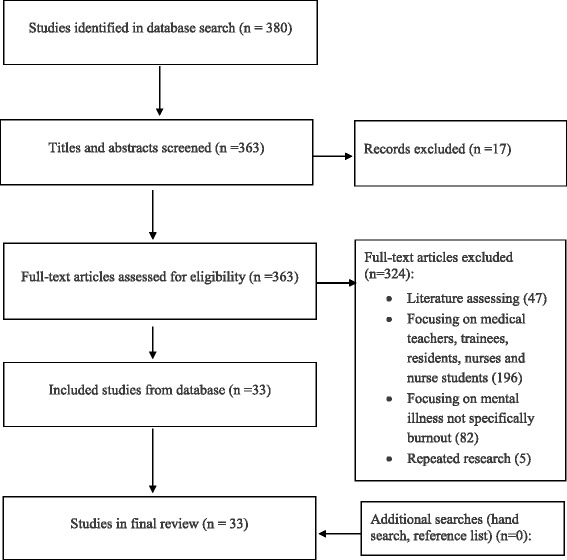
Flow diagram of the search and selection process

Using the search strategy described above, 380 references were retrieved, including 6 articles from MEDLINE, 157 articles from the China Academic Journals Full-text Database, 99 articles from the Chinese Science Citation Database and 118 articles from Wanfang Data Resource System. Thirty-three studies were included (Fig. 1).

### Characteristics of included studies

The main characteristics of the included studies are listed in Table 1. The range of sample size was between 77 and 1402. There was a total of 14,774 participants across all of the studies. The year of the studies included were from 2008 to 2015. Among 33 studies, seven studies divided participants into two or three groups to compare; 10 studies recruited students from more than two institutions. There were 25 studies analysing the present situation, seven studies were retrospective control studies and only one prospective cohort study (see Table 1). There was no randomized controlled experiment study design among the articles. Seven were non-randomized two group studies while one was a single group pre and post-test design. The remainder were single group cross-sectional or single group post-test only.

### Study quality

Among the the 33 included studies, the MERSQI score range was 8–13. The mean total MERSQI score was 11.3. Response rate of all the studies were over 75%. Data analysis of all the articles included were appropriate for study design and type of data. However, the outcome of the articles are mostly satisfaction, attitudes, perceptions, opinions and general facts according to MERSQI. Only two articles developed knowledge or skills as outcome based on the study [30, 31].

### Review findings

Included studies reported high levels of burnout amongst medical students in China, with over 40% of students in most studies identified as having more than moderate levels of burnout [32–37].Among the 33 studies included, only five studies solely targeted graduate medical students, with three further studies that targeted graduate medical students in addition to interns and undergraduate medical students [33, 38–43]. The remaining 25 studies targeted undergraduate medical students.

i. Demographic factors

Gender, age and whether the student was from an urban or rural setting were all identified as significant predictors in some studies, but findings were not consistent across the included studies. A total of eight studies reported gender as a significant predictor of burnout or its at least one of its constructs, with males experiencing a greater degree of suffering than females [33, 35, 37, 41, 42, 44–47]. Of the studies identified, two found that scores for emotional exhaustion in males were significantly higher than in females [41, 42]. Another four studies found that males’ scores for depersonalization were also significantly higher than in females [35, 44, 45, 47]. In contrast, Yang et al. (2011), reported significantly lower scores on personal accomplishment in males than in females and two further studies found no significant difference in the total burnout score between males and females [30, 44, 48].

Overall burnout scores among different grades of medical students varied significantly in six studies [30, 36, 45, 46, 48, 49]. All identified that more senior students suffered greater burnout, with third and fourth year students reporting significantly higher scores than first and second year students [30, 36, 45, 46, 48]. Only one study did not report any significant differences between overall burnout scores between different grades of students [47]. Related to this, four studies reported a positive correlation between burnout and the students’ sense of continuing commitment to the medical profession or study pressure experienced [31, 40, 50, 51]. A significant negative correlation between students motivation to accomplish and burnout was reported in two further studies, although a third found no such association [52–54].

The extent of emotional exhaustion and depersonalization among medical students significantly increased with age in one study [55]. Whilst a second study identified that the level of emotional exhaustion and depersonalisation significantly increased with age, no significant difference in the overall burnout score was identified between different age groups [39].

A statistically significant difference was identified in one study in the overall burnout scores of medical students from different areas (village, town and city); students who came from a village had higher learning burnout scores than students who came from a city or township [30]. An additional study reported that whilst there was no significant difference in overall burnout scores of medical students from rural or urban settings, medical students from rural areas had a significantly lower score than those from cities in the dimension of personal accomplishment [41]. Another two studies reported no significant difference in overall burnout scores or any dimension of burnout between those in different areas. [33, 45]

ii. Social factors

A total of three studies explored the association between social support and burnout using the Social Support Rate Scale (SSRS) [35, 39, 42]. Two of these studies found that the emotional exhaustion and depersonalization dimensions of burnout among medical students were significantly negatively correlated with social subjective support and the utilisation level of social support [39, 42]. The third study found that all the three dimensions of burnout were significantly negatively correlated with social subjective support such as family, friends and organisation [35]. Social support and self-efficacy were identified as negatively associated with burnout in two further studies [47, 56]. One of these explored social support in terms of the interpersonal relationships and learning atmosphere in dormitories in which medical students lived [47]. The results showed that interpersonal relationships and learning atmosphere were negatively correlated with dimensions of burnout (exhaustion and depersonalisation), which meant that the better interpersonal relationships within the dormitories was able to reduce the burnout rate.

iii. Psychological factors

Of the studies identified, three reported associations between burnout and other psychological constructs, indicating that those experiencing poorer mental health overall were also more likely to suffer burnout [30, 32, 57]. Zhang et al. (2011) reported a significant positive correlation between the dimensions of exhaustion and depersonalization and a student’s general psychological condition (using the SCL-90 symptoms self-evaluation scale) [57]. There was a significant negative correlation between the burnout dimension of personal achievement and a student’s psychological condition. Zhu et al. (2012) found that increased emotional exhaustion and a low sense of personal achievement were significantly positively associated with a student’s psychological condition (SCL-90 symptoms self-evaluation scale) [32]. In the third study, overall burnout and each of its constructs were independently positively associated with higher stress scores (*r* = 0.184~0.349) [30]. Overall burnout scores were negatively correlated with coping styles that focused on problem solving and asking for help(*r* = −0.383~ − 0.255) but positively correlated with coping styles that included self-blame, fantasy, retreat and rationalisation (*r* = 0.234~0.421) [30].

## Discussion

Of the 33 included studies on burnout published in Chinese, most focused on demographic factors. More specifically, being male, experiencing poorer psychological functioning and being a more senior medical student were found to correlate with increased burnout. While the demographic factors were well substaintiated substantiated, our review shows a lack of research into other contributing factors. This is despite growing evidence to highlight the valuable role of contextual factors in interventions to reduce burnout [19].

The few studies in this review that explored contextual or social factors indicate these are important in the Chinese context. Findings that show both lack of social support (as a contributing factor to burnout) and a supportive environment in living quarters (as a mediating factor), affect rates of burnout flag the potential importance of context in both prevention and remediation strategies [35, 38, 39, 42]. Additional evidence regarding the contextual and social factors that promote or protect medical students from burnout is critical in order to develop contextually relevant and effective prevention strategies for this group.

Few studies targeted graduate students (those who already have their degree, also referred to as postgraduate). Greater exploration of the unique factors affecting graduate medical students are necessary due to the different curriculum, the substantial proportion of medical students in this group and their learning environment [39, 41]. The average age of enrollment for graduate medical students is generally higher then undergraduate students - this population may therefore encounter different contextual and social factors and pressures that influence their experience of burnout. Warranting further study of this specific group.

### Implications

Three implications emerge from our review. The literature reviewed demonstrates that burnout is a challenge for medical students in China, but there is a lack of studies into factors beyond demographic variables that might predict increased burnout. This in turn means that the scope for evidence-based intervention development is limited. Secondly, even though demographic studies dominate, comparatively little is known about burnout in graduate medical students who have a markedly different demographic profile from their undergraduate counterparts [58]. Lastly, as research into interventions to prevent burnout is limited in China, there may be value assessing international interventions identified as effective within Chinese settings [19].

### Limitations

Limitations apply to this study both in terms of the included studies and the review methods used. Non-published and non-empirical work were not included and as such may have led to the omission of relevant perspectives [59]. Bibliographic databases vary in their levels of specificity and sensitivity, impacting the number of articles returned and those that are considered relevant to the search terms [60]. Several databases in addition to manual searching were used to broaden coverage but relevant material may have been omitted. With the limitation number of the included articles, all the articles scored by MERSQI ranging from eight to13 were accepted to maximize the information. However, it might affect the quality of the conclusion.

## Conclusions

The review findings highlight recognition of the problem of burnout amongst medical students in China, and that medical students are at risk of burnout. The evidence available is currently limited which is a barrier to developing effective, context-specific interventions. Additional studies that explore the contextual and social factors affecting burnout rates, the experiences of burnout in graduate students and that test existing interventions identified as effective in other settings in a Chinese context are needed.

## Abbreviations

CY: Cynicism
EX: Exhaustion
LRS: Lian rong survey
MBI: Maslach burnout inventory
MBI-GS: MBI – general survey
MBI-HSS: MBI – human services survey
MBI-SS: MBI – student survey
MERSQI: Medical education research study quality instrument
PE: Professional efficacy
PSMS: Postgraduate student mentor scheme
SCI: Science citation index CY
SCL-90: Symptom checklist-90
SPRF: Survey with the possible related factors
